# Does curve magnitude in adolescent idiopathic scoliosis (AIS) affect frequency and quality of sport participation? A feasibility study

**DOI:** 10.1186/s40814-020-00745-4

**Published:** 2021-01-12

**Authors:** Michael Youssef, John Soliman, Sarah Burrow, Waleed Kishta, Nicole Simunovic, Andrew Duong, Olufemi R. Ayeni, Devin Peterson

**Affiliations:** 1 Faculty of Health Sciences, McMaster University, Hamilton, ON Canada; 2 Division of Orthopaedic Surgery, Department of Surgery, McMaster University, Hamilton, ON Canada; 3 Department of Health Research Methods, Evidence and Impact, McMaster University, Hamilton, ON Canada

**Keywords:** Adolescent idiopathic scoliosis, Curve size, Sport participation, Health-related quality of life, Shortness of breath

## Abstract

**Background:**

This pilot study explores whether large adolescent idiopathic scoliosis (AIS) curves (≥ 45°) lead to decreased frequency and quality of sport participation, lower health-related quality of life (HRQL), and more pronounced shortness of breath (SOB) as compared to smaller curvatures (< 45°).

**Methods:**

Patients were divided into two groups based on their spinal curvature: Cobb angle < 45° (*n* = 31) and ≥ 45° (*n* = 21). We assessed feasibility outcomes including agreement to be approached, participation, recruitment rates and missing data. All participants completed five questionnaires to assess the frequency and quality of sport participation, HRQL and SOB outcomes. Estimates of effects 95% confidence intervals (CIs) were reported.

**Results:**

This study enrolled 52 surgically untreated AIS patients between the ages of 10 and 18 (44 females, 8 males, mean age = 14.60). All feasibility threshold criteria were successfully met (100% agreement to be approached, 100% participation with *n* ≥ 12 in each group, and 94.2% of patients without missing data). AIS patients with large curvatures (≥ 45°) trended towards decreased frequency and quality of sport participation, more pronounced SOB and worse HRQL outcomes, as compared to patients with smaller curve sizes.

**Conclusion:**

The study findings show that a study addressing sport participation in the setting of AIS is feasible. The size of curvature in AIS may have an impact on sport participation, HRQL and SOB, but larger studies are required.

## Key messages regarding feasibility

- Many uncertainties exist regarding the feasibility of conducting a large-scale study exploring the magnitude of AIS curve size on sport participation and health outcomes. Firstly, little research has been published on this research question, and thus, there is a lack of scoliosis-specific questionnaires that measure the outcomes of interest. This raises concerns regarding the feasibility of using non-scoliosis questionnaires to assess the study outcomes. Secondly, the study uses many questionnaires which can be time-consuming. Thus, it is unclear whether patients would be willing to participate in the study or if results would yield substantial missing data. Lastly, the feasibility of recruiting untreated AIS patients with curves above 45° needs to be determined in the clinical setting, considering that such patient population would rarely go untreated as they progress to such severity. Determining recruitment rates is important to know whether it is realistic to recruit large sample sizes in this patient population before committing resources to a large-scale study.
- Key feasibility findings include 100% agreement to be approached, 100% patient participation, 52 patients recruited over 2 months exceeding the success threshold criteria for recruitment rates, 94.2% of patients completed the study questionnaires and measurements without missing data and in less than 15 min.
- Findings from this study support the feasibility of recruiting a large sample size and conducting a large-scale powered study to further explore the research question. Furthermore, results indicate that the study procedures and tools are feasible to use in the clinical setting and can be used to explore the severity of scoliosis and its impact on sport participation and health outcomes.

## Background

Adolescent idiopathic scoliosis (AIS) constitutes approximately 90% of all scoliosis cases in children and youth [1]. AIS literature has primarily focused on the impact of the disease on respiratory and health-related quality of life (HRQL) outcomes. However, there has been a paucity of literature regarding its impact on sport participation, an integral part of adolescents’ lifestyle, specifically, how AIS affects the frequency and quality of sport engagement [2].

Curve magnitude is of particular importance in AIS as it dictates the severity of the condition. A curve size of 45° is a standard threshold that categorizes mild/moderate curves from severe ones [3]. Studies conducted on AIS patients have demonstrated that a large curvature is implicated in increasing shortness of breath (SOB) and worsening HRQL outcomes such as pain and self-image concerns [4–8]. However, other studies have shown that curve magnitude had no effect on HRQL [9, 10].

The benefits of sport participation in adolescents are many including developing friendships and discipline and collaborating with others. The ability of patients with AIS to participate in sport has been very underreported. With regards to sport participation, one study found no relationship between curve magnitude and sport frequency in untreated AIS patients, and there are no studies on the quality of sport participation [2].

The purpose of this study is to determine the feasibility of studying sport participation in patients with severe AIS and suggest potential modifications to the procedures used in order to more fully capture this population in future studies. Additionally, this study explores whether AIS patients with curve sizes ≥ 45° tend to experience decreased frequency and quality of sport participation, lower HRQL, and more pronounced SOB during activity as compared to patients with smaller curves.

## Methods

The aim of this pilot study is to assess the feasibility of studying sport participation in patients with severe AIS and to explore potential trends regarding the impact of AIS curve size on sport participation, HRQL and SOB outcomes.

### Study population

This cross-sectional pilot study enrolled 52 AIS patients who had not had deformity surgery (44 females, 8 males) between the ages of 10 and 18 (mean age = 14.6) over a period of 2 months. Patients were divided into two groups based on their spinal curvature: Cobb angle < 45° (*n* = 31) and ≥ 45° (*n* = 21). Cobb angles were calculated using the largest of the proximal thoracic (PT), main thoracic (MT) or thoracolumbar/lumbar (TL-L) components [9]. All curves were considered if they were at least 10° or more in magnitude and were then classified as proximal thoracic (PT), main thoracic (MT) or thoracolumbar/lumbar (TL/L).

### Setting and recruitment

Patients were recruited from the Orthopedic clinic at McMaster Children’s Hospital in Hamilton, Ontario, Canada. Ethics approval was obtained from the Hamilton Integrated Research Ethics Board (HiREB #3989). Eligible participants were screened during their regular clinic visit by their orthopaedic surgeon and parents were asked by the clinic staff for verbal consent prior to their child being approached for participation. The team then explained the study rationale and obtained informed consent for participation.

### Eligibility criteria

Inclusion criteria included patients between the ages of 10 and 18 who were diagnosed with AIS and had not undergone surgical management of their deformity. Participants had to be able to speak, understand, and read the language of the clinical site. As minors, participants had to provide both assent and parental consent. Patients were excluded if they had any of the following as their primary diagnosis: kyphosis, spondylolisthesis, spina bifida or any developmental disabilities that limit the participant’s ability to understand the questionnaires.

### Feasibility outcomes

The following a priori thresholds were used to assess feasibility measures:

1. Agreement to be approached: more than 80% of eligible patients agree to hear about the study from the research team.
2. Agreement to participate: more than 80% consent to participate from all patients who agreed to hear about the study.
3. Recruitment rates: 24 subjects recruited within 2 months (12 in each group). A sample size of 12 participants per group is a common convention used for pilot studies if there is no prior information to base a sample size on [11].
4. Missing data: more than 80% of participants, who consent for participation, complete all questionnaires without missing data.

All participants were timed as they complete the questionnaires to provide more accurate information regarding the time commitment required by patients.

### Questionnaires (Additional file 1: Appendix)

#### Assessment of sport participation frequency—sport score

A Sport Score questionnaire as described by Noyes et al. [12] was used for sport participation [12]. This questionnaire explores the frequency of engaging in sport and the intensity of the sport played. A maximum score is 100 indicating 4–7 days/week participation in strenuous sports such as basketball or soccer.

#### Assessment of sport participation quality—PODCI

Items relevant to the quality of sport activity from the self-reported version of the Pediatric Outcomes Data Collection Instrument (PODCI) were used to determine the quality of sport participation. The score for each item ranges from 1 to 4 with lower numbers indicating higher sports functioning. A total mean score was calculated for each participant by adding scores from individual items [13].

#### Assessment of respiratory symptoms—MRC and UCDQ

Two respiratory questionnaires, the Medical Research Council (MRC) Breathlessness scale and the University of Cincinnati Dyspnea Questionnaire (UCDQ), were used to determine whether SOB was encountered during sport participation [14, 15]. When scoring MRC and UCDQ, patients select a grade from 1 to 5 to describe their SOB, with lower grades indicating better respiratory function [14–16]. With regards to UCDQ, only 4 relevant items were adopted from the original questionnaire and a mean score was calculated. When patients selected “not interested” for any of the activities listed on the UCDQ questionnaire, the score for the individual item was not factored in calculating the average score.

#### Assessment of HRQL—SRS-22r

The Scoliosis Research Society (SRS-22r) questionnaire, a reliable and valid assessment tool, was used to explore the overall HRQL score [16]. Additionally, subdomain scores including function, pain, self-image, mental health and satisfaction scores were reported [17]. The scoring system of the SRS-22r is published elsewhere [17].

Cobb angles were measured by one of the participating paediatric orthopaedic surgeons, and potential confounders including physiotherapy, bracing and body mass index (BMI) were also recorded.

### Descriptive data analysis

Percentages were calculated for all feasibility outcome measures. Average means, standard deviations (SD) and estimates of effects (95% CIs) were reported for all patient-reported variables and outcomes.

## Results

### Feasibility outcomes

All a priori feasibility measures outlined above were successfully met. All 59 patients approached by the clinic staff agreed to hear about the study from the research team (100% agreement to be approached). Of those who were approached by the research team and had their eligibility confirmed (*n* = 52), all consented to participate in the study (100% participation). Recruitment rate exceeded the success threshold, where a total of 52 participants were recruited over a period of 2 months (*n* = 31 for the < 45° group; *n* = 21 for the ≥ 45°group). Of the 52 patients recruited, 49 patients answered all the questionnaires and had complete measurements without any missing data (94.2%), thereby exceeding the success threshold. With regards to timing, 94.2% of patients completed the study in 15 min or less with an average time of 11.71 ± 3.10 min.

### Participant recruitment

**Fig. 1:**
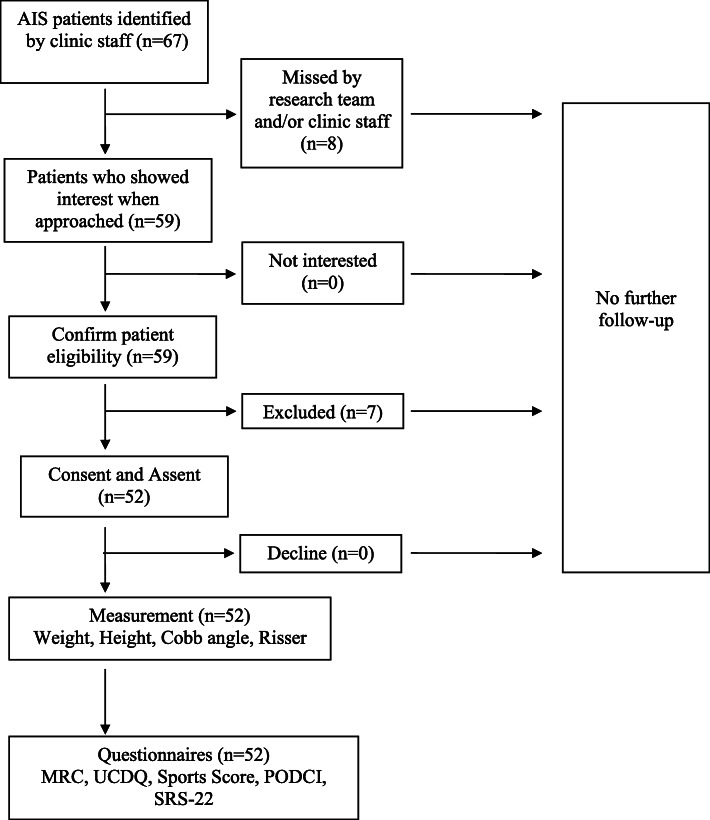
Patient recruitment model

The process by which participants were recruited is outlined in Fig. 1. Over a period of two months, 67 untreated AIS patients were identified by the clinic staff. A total of eight eligible patients were missed in this study and were never approached by the study team. Of the 59 patients who were approached, seven patients were excluded after application of the inclusion/exclusion criteria. Reasons for exclusion were a diagnosis of spondylolisthesis (*n* = 1), spina bifida (*n* = 2), congenital scoliosis (*n* = 1) and developmental disabilities (*n* = 3).

### Baseline demographic data

Table 1 reports demographic characteristics of the study population. Participants were predominantly female (female = 48, male = 8) with a mean age of 14.6. This is representative of the AIS population demographic, where females generally have a higher incidence and severity of the disease compared to males, with literature suggesting a 7.2:1 female to male ratio in curves > 40° [1]. The average Cobb angle was 23.34° in the < 45° group and 56.9° in the ≥ 45° group. Nineteen patients of the ≥ 45° group had an MT curve size ≥ 45°.

**Table 1:** Study population demographics

	Cobb angle < 45° (*n* = 31)	Cobb angle ≥ 45° (*n* = 21)	Overall (*n* = 52)
Age, years (mean ± SD)	14.16 ± 1.55	15.24 ± 1.76	14.60 ± 1.71
Gender, *n* (male/female)	5/26	3/18	8/44
Weight, kg (mean ± SD)	53.32 ± 9.25	60.88 ± 15.59	56.43 ± 12.69
Height, cm (mean ± SD)	164.16 ± 10.16	165.84 ± 9.61	164.84 ± 9.88
PT angle (°) (mean ± SD)	11.02 ± 7.27	29.65 ± 11.73	18.33 ± 12.98
MT angle (°) (mean ± SD)	17.90 ± 11.19	54.88 ± 12.72	33.13 ± 21.80
TL/L angle (°) (mean ±, SD)	18.37 ± 10.27	40.30 ± 10.52	27.23 ± 14.95
Cobb angle (°) (mean ± SD)	23.34 ± 9.46	56.9 ± 10.15	36.89 ± 19.22
Risser sign	3.56 ± 1.73	3.71 ± 1.51	3.63 ± 1.63

*PT* proximal thoracic, *MT* main thoracic, *TL/L* thoracolumbar/lumbar

Fourteen patients (*n* = 8 in the < 45° group; *n* = 6 in the ≥ 45°group) indicated their use of a brace for an average of 15.75 h daily, with no observable differences between groups. Additionally, 15 patients reported receiving one or more treatment(s) for their back, including physiotherapy (*n* = 9), osteopathy (*n* = 2), chiropractic (*n* = 3), acupuncture (*n* = 1) and stretches (*n* = 2). Physiotherapy was the most common intervention with an average of 3.28 h/week, and no major differences between groups. The mean BMI for the study sample was 20.69, and there were no major differences between groups (19.70 for the < 45° group; 22.10 for the ≥ 45°group).

### Patient-reported outcomes

#### Frequency and quality of sport participation

Table 2 shows the results of the sport outcomes explored in this study. Data suggests a trend towards greater curvature being associated with both decreased frequency and quality of sports participation. On the PODCI questionnaire, 9 patients described not participating in sports for the reasons of dislike of sports (*n* = 8) or pain while playing sports (*n* = 1; in the ≥ 45°).

**Table 2:** Sports outcomes

	Below 45° group Mean ± SD (95% CI)	Above 45° group Mean ± SD (95% CI)
Frequency of sport participation (Sport Score)	74.68 ± 25.36 (65.8–83.6)	69.05 ± 24.01 (58.8–79.3)
Quality of sport participation (PODCI)	1.27 ± 0.39 (1.13–1.41)	1.48 ± 0.49 (1.27–1.69)

#### Sob

The results regarding SOB as measured by both the MRC and UCDQ questionnaires are shown in Table 3. Patients with greater curvatures trended towards experiencing more SOB during sport participation.

**Table 3:** Shortness of breath (SOB) outcomes

Questionnaire	Below 45° group Mean ± SD (95% CI)	Above 45° group Mean ± SD (95% CI)
MRC	1.26 ± 0.44 (1.1–1.42)	1.52 ± 0.68 (1.23–1.81)
UCDQ	2.33 ± 0.89 (2.01–2.65)	2.55 ± 1.05 (2.08–3.02)

*MRC* Medical Research Council, *UCDQ* University of Cincinnati Dyspnea Questionnaire

#### HRQL

HRQL outcomes were assessed using the SRS-22r questionnaire, and total and subdomain scores are shown in Table 4. Generally, there were trends with greater curvature being associated with increased pain and self-image concerns as well as decreased function and satisfaction with treatment measures. Furthermore, there was a generally higher SRS22 total score in the below 45° group reflecting better HRQL as compared to patients with more severe curvatures.

**Table 4:** SRS-22r total and subdomain scores

Domain	Below 45° group Mean ± SD (95% CI)	Above 45° group Mean ± SD (95% CI)
SRS22r pain	4.28 ± 0.64 (4.06–4.5)	4.26 ± 0.69 (3.96–4.55)
SRS22r function	4.52 ± 0.44 (4.36–4.67)	4.32 ± 0.65 (4.04–4.6)
SRS22r self-image	4.01 ± 0.62 (3.79–4.23)	3.60 ± 0.84 (3.24–3.96)
SRS22r mental health	3.84 ± 0.79 (3.56–4.12)	3.85 ± 0.94 (3.45–4.25)
SRS22r satisfaction	4.05 ± 0.77 (3.78–4.32)	3.98 ± 0.73 (3.67–4.29)
SRS22r total	4.15 ± 0.49 (3.98–4.32)	4.00 ± 0.66 (3.72–4.28)
SRS22r total (w/o satisfaction)	4.16 ± 0.51 (3.98–4.34)	4.01 ± 0.68 (3.72–4.30)

*SRS22* Scoliosis Research Society

## Discussion

This is a pilot study designed to explore the feasibility of conducting a larger study that assesses the impact of curve size in AIS patients on the frequency and quality of sport participation. The most significant finding was that all feasibility criteria were successfully met, supporting the efficacy of the study tools and protocols. The feasibility component of the study suggests that larger-scale trials can be performed as patients were interested in participating and the burden of time to complete the questionnaires is very reasonable. Generally, data suggests a trend towards greater curvatures being associated with decreased frequency and quality of sport participation. This suggests that AIS patients with a large curve size (≥ 45°) might face more functional limitations when participating in sports than those with lesser curve magnitude.

A Cobb angle of 50° or greater at skeletal maturity has been found to be a predictor of decreased pulmonary function and patients with curves between 60 and 100° have been found to have a total lung capacity 68% of normal [18, 19]. In the current study, patients with larger curves appear to have more SOB on the MRC questionnaire and similar to findings from Danielsson et al. [20]. They also had similar results on the UCDQ questionnaire, further supporting the association between curve size and SOB.

The relationship between curve magnitude and HRQL outcomes, in AIS literature, has largely been inconclusive with conflicting results. A study by Climent et al. showed that curve magnitude is associated with worse pain, function, self-image, mental health, satisfaction and total SRS scores [21]. However, other studies have shown no correlations between curve size and the aforementioned health-related outcomes [22–24]. In our pilot study, there were trends towards more significant pain and self-image concerns with lower functioning and satisfaction in the greater curvature group. This makes the results more consistent with studies like that by Climent et al. but larger more powered studies are needed to further explore those important outcomes.

This pilot study has several strengths. First, it is one of few studies addressing a very important part of an adolescent’s life, sport participation. Second, the recruitment rate suggests that there is interest in both patients and caregivers to explore issues relating to AIS and sport participation. The recruitment rate was robust and enables planning for future properly powered studies addressing this topic. Part of the success in recruitment stems from the multidisciplinary team involved in both recruitment and patient interaction, as well as the limited burden posed by the study procedures. With an overall prevalence of 0.5–5% [1], there would be little problem in performing a large-scale study of this population. In fact, AIS is the most common spinal deformity seen by primary care doctors and spine surgeons [25], thus ensuring a continued high recruitment rate for a larger-scale study. Furthermore, many patients completed the study tools during their normal wait-time, avoiding any extra time commitment beyond their routine care. The standard deviation (SD) of the Sport Score questionnaire was calculated to be 24.7 which is an important parameter for calculating a sample size for future powered studies; however, other studies are needed to find the minimal clinically important difference (MCID) of this tool. Both parameters are required to calculate an appropriate sample size for future powered studies exploring the frequency of sport participation as a primary outcome.

The study has several limitations to consider. First, the research team was not able to be present due to other commitments for a total of eight patients who were therefore not recruited for the study. Recruitment can be further improved by ensuring a larger number of research staff who can cover more of the clinic’s working hours. Second, some of the questionnaires utilized are not spine-specific tools, and thus, there might be concerns about their validity in the context of AIS. However, the Sport Score questionnaire has been used by Parsch et al. [2] in their scoliosis study. Finally, this feasibility study only reports “trends” and potential associations between curve size and sports and health-related outcomes. Larger studies will be required to further explore those associations and draw more conclusive findings.

## Conclusion

This study suggests that larger-scale trials can be reasonably performed as patients were interested in participating and the burden of time to complete the questionnaires is reasonable. The preliminary findings show trends suggesting that a large spine curvature (≥ 45°) may be associated with decreased frequency and quality of sport participation, increased SOB during activities and worse HRQL outcomes. However, the inherent limitations of pilot studies require that future high-powered research be conducted to further explore this research question.

## Supplementary Information

**Additional file 1:** Questionnaires (Appendix 1).

## Data Availability

The datasets used and/or analysed during the current study are available from the corresponding author on reasonable request.

## Abbreviations

AIS: Adolescent idiopathic scoliosis
HRQL: Health-related quality of life
SOB: Shortness of breath
PT: Proximal thoracic
MT: Main thoracic
TL-L: Thoracolumbar/lumbar
PODCI: Pediatric Outcomes Data Collection Instrument
MRC: Medical Research Council
UCQD: University of Cincinnati Dyspnea Questionnaire
SRS: Scoliosis Research Society
BMI: Body mass index
SD: Standard deviation
CI: Confidence intervals
MCID: Minimal clinically important difference
