# Supplementary material for: Does curve magnitude in adolescent idiopathic scoliosis (AIS) affect frequency and quality of sport participation? A feasibility study
Source: Pilot Feasibility Stud. 2021 Jan 12;7:26. doi: 10.1186/s40814-020-00745-4 (PMC7802301; doi:10.1186/s40814-020-00745-4)
Supplement: Supplementary file 1 — Additional file 1: Questionnaires (Appendix 1). [file 40814_2020_745_MOESM1_ESM.docx]

**APPENDIX**

1. **Sport Score Questionnaire**

**
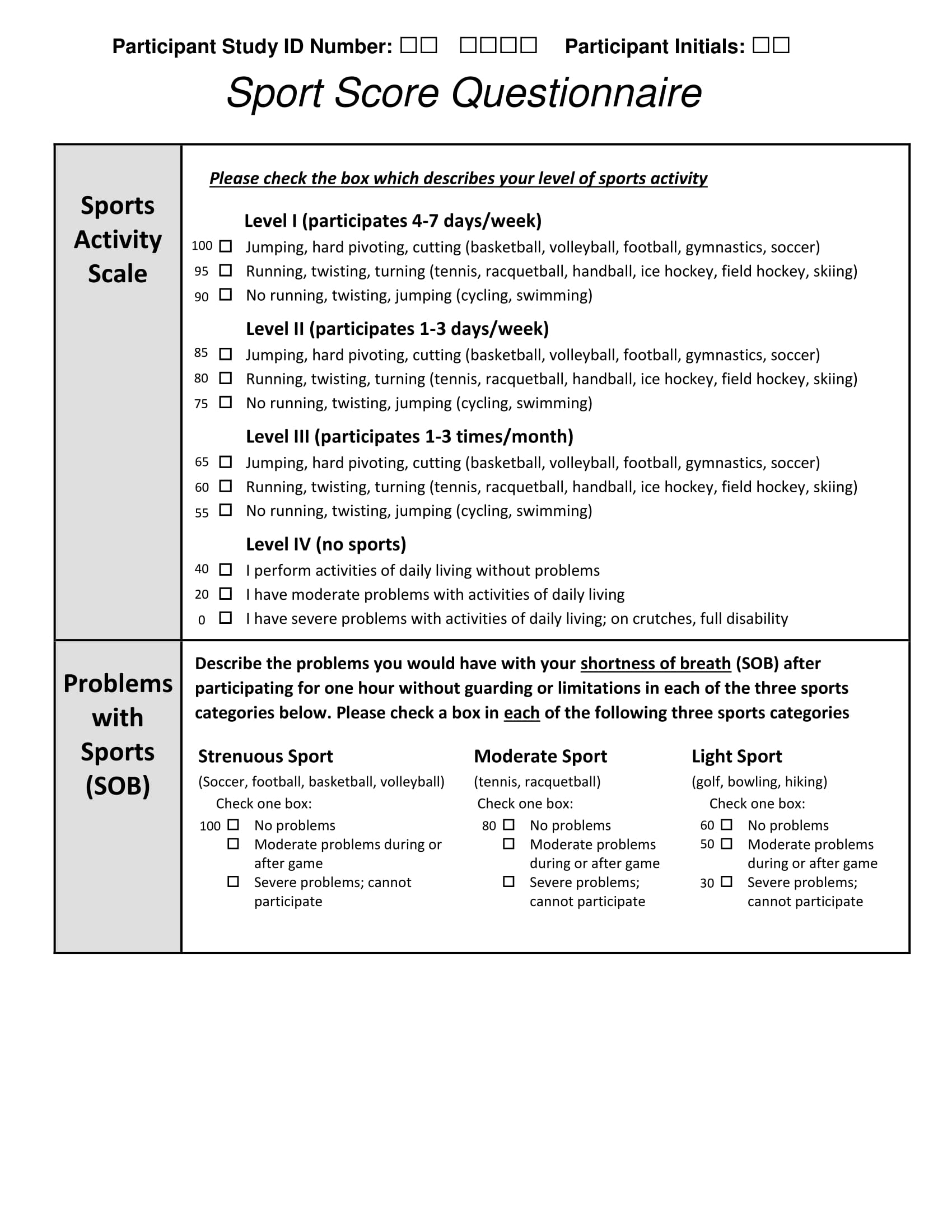
**

1. **PODCI**

During the **last week**, has it been easy or hard for you to: (choose one response per line)

|  | ☐ Easy | ☐ A little hard | ☐ Very hard | ☐ Can’t do at all |
| --- | --- | --- | --- | --- |
| **1**. Run short distances? |  |  |  |  |
| **2**. Bicycle or tricycle? |  |  |  |  |

**3**. Can you participate in **recreational outdoor activities** with other kids the same age? (For example: bicycling, skating, hiking, jogging) (choose one response.)

☐ Yes, easily ☐ Yes, but a little hard ☐ Yes, but very hard ☐ No

If you answered “no” to question 3, was your activity limited by: (choose all that apply)

*☐ Pain? ☐ General health? ☐ Doctor or parent instructions? ☐ Fear the other kids won't like you? ☐ Dislike of recreational outdoor activities?* *☐ Activity not in season?*

**4.** Can you participate in **pickup games or sports** with other kids the same age? (For example: tag, dodgeball, basketball, softball, soccer, catch, jump rope, touch football, hop scotch) (choose one response)

☐ Yes, easily ☐ Yes, but a little hard ☐ Yes, but very hard ☐ No

If you answered “no” to question 4, was your activity limited by: (choose all that apply)

*☐ Pain? ☐ General health? ☐ Doctor or parent instructions? ☐ Fear the other kids won't like you?* *☐ Dislike of pickup games or sports?*  *☐ Activity not in season?*

**5**. Can you participate in **competitive level sports** with other kids the same age? (For example: hocket, basketball, soccer, football, baseball, swimming, running [track or cross country], gymnastics, or dance) (choose one response)

☐ Yes, easily ☐ Yes, but a little hard ☐ Yes, but very hard ☐ No

If you answered “no” to question 5, was your activity limited by: (choose all that apply)

*☐ Pain? ☐ General health? ☐ Doctor or parent instructions? ☐ Fear the other kids won't like you?* *☐ Dislike of competitive level sports?*  *☐ Activity not in season?*

1. **MRC Breathlessness Scale**

**
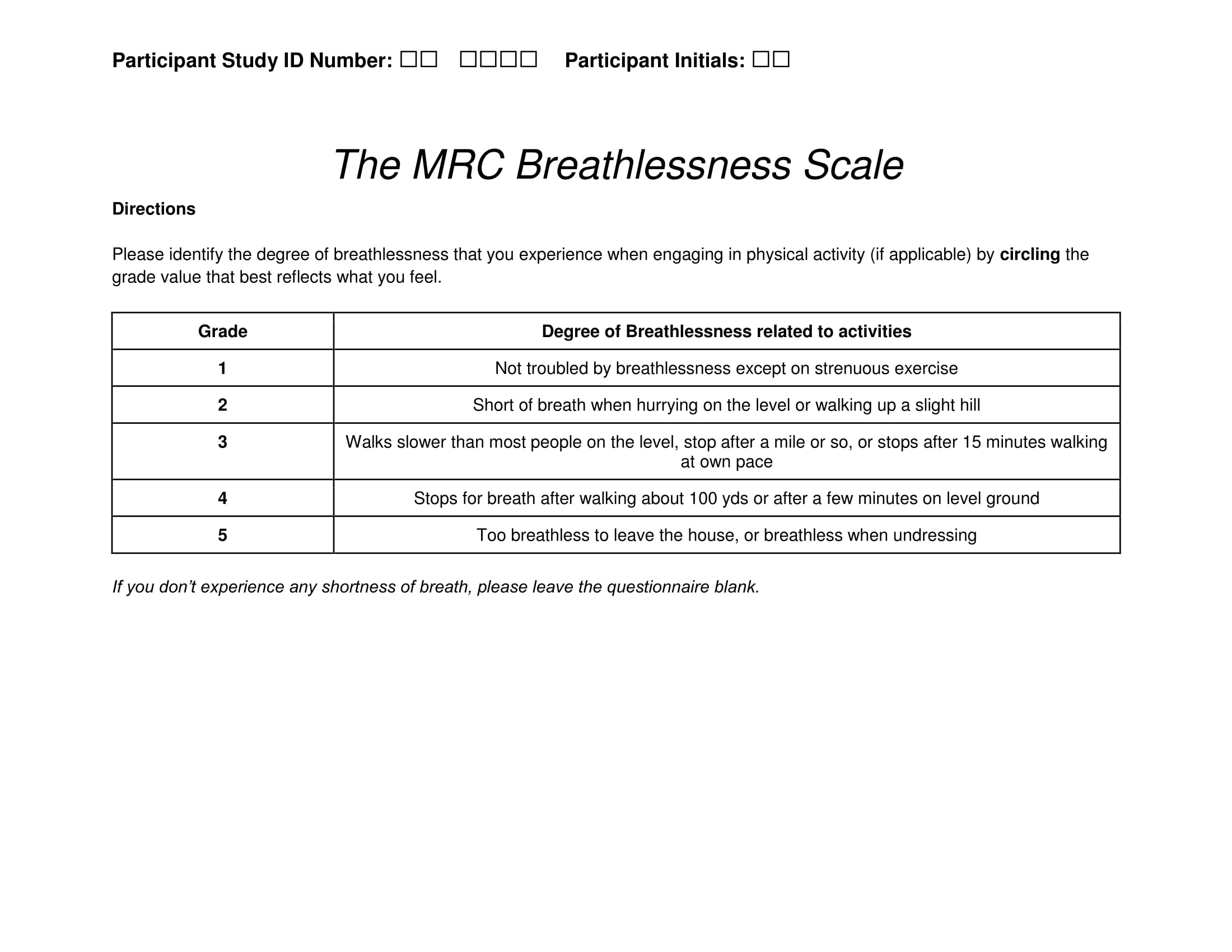
**

1. **UCDQ**


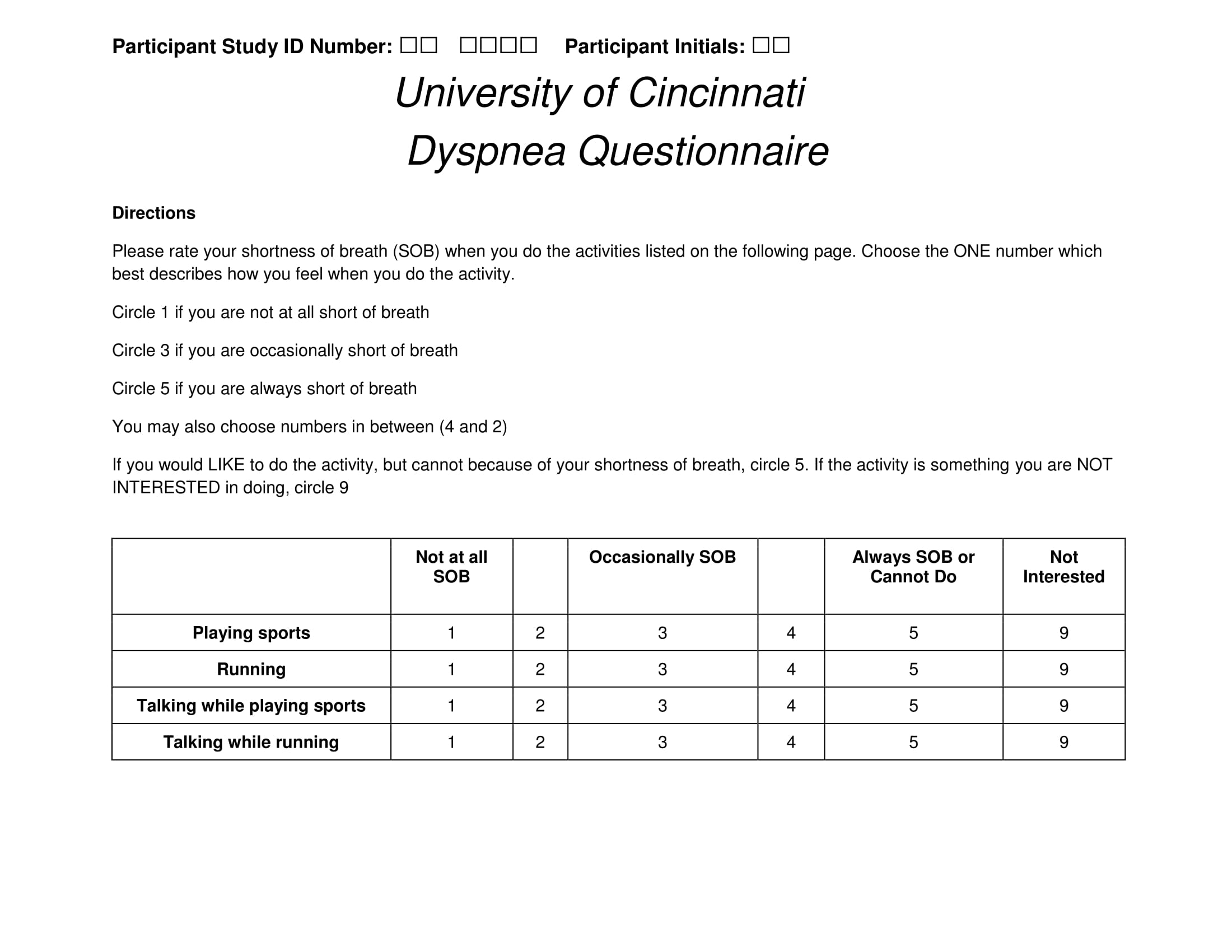


1. **SRS-22r** (available from: <http://www.srs.org/UserFiles/file/outcomes/srs-22_sample.pdf>)
